# Supplementary material for: Weyl Fermion magneto-electrodynamics and ultralow field quantum limit in TaAs
Source: Sci Adv. 2022 Jan 14;8(2):eabj1076. doi: 10.1126/sciadv.abj1076 (PMC8759752; doi:10.1126/sciadv.abj1076)
Supplement: Supplementary file 1 — Sections S1 to S8 Figs. S1 to S8 References [file sciadv.abj1076_sm.pdf]

Science Advances
AAAS

Supplementary Materials for

Weyl Fermion magneto-electrodynamics and ultralow field quantum limit in TaAs

Zhengguang Lu, Patrick Hollister, Mykhaylo Ozerov, Seongphill Moon, Eric D. Bauer, Filip Ronning, Dmitry Smirnov, Long Ju*, B. J. Ramshaw*

*Corresponding author. Email: bradramshaw@cornell.edu (B.J.R.); longju@mit.edu (L.J.)

Published 14 January 2022, *Sci. Adv.* **8**, eabj1076 (2022)
DOI: 10.1126/sciadv.abj1076

This PDF file includes:

Sections S1 to S8
Figs. S1 to S8
References

Supplementary Information: Weyl Fermion Magneto-Electrodynamics and Ultra-low Field Quantum Limit in TaAs

1. Low energy model and inter-LL transitions.

A minimal two-band model that describe the Weyl nodes can be written as *(12)*

$$H(k) = a(k_w^2 - k^2)\sigma_z + \hbar v_F(k_x\sigma_x + k_y\sigma_y), \tag{1}$$

where σ are the Pauli matrices, k is the wavevector, v_F is the Fermi velocity, and *a* is a fitting parameter. This two band Hamiltonian gives a global description of the topological properties of a pair of Weyl nodes with opposite chirality *(12)*. The dispersion relations of these two energy bands are:

$$E(k)_\pm = \pm\sqrt{(a(k_w^2 - k^2))^2 + \hbar^2 v_F^2(k_x^2 + k_y^2)}\,. \tag{2}$$

At k_x, k_y=0, the two bands intersect at (0, 0, $\pm k_w$), which are the Weyl points. If we apply a magnetic field in *z*-direction (along the direction of Weyl point separation), the electronic bands are quantized into a set of Landau levels dispersing along k_z. The dispersion relation is

$$E(k_z)^0 = \frac{a}{l_B^2} - ak_w^2 + ak_z^2\,, \text{N=0} \tag{3}$$

$$E(k_z)^N = \frac{a}{l_B^2} \pm \sqrt{(ak_w^2 - ak_z^2 - \frac{2a}{l_B^2}N)^2 + \frac{2\hbar^2 {v_F}^2}{l_B^2}N}\,, \text{N}\geq 1, \tag{4}$$

where N is the Landau index and $l_B \equiv \sqrt{\hbar/|eB|}$ is the magnetic length. At $k_z=\pm k_w$, the dispersion relation for N≥1 reduced to

$$E(\pm k_w)^N = \frac{a}{l_B^2} \pm \sqrt{\frac{4a^2}{l_B^4}N^2 + \frac{2\hbar^2 {v_F}^2}{l_B^2}N}\,. \tag{5}$$

The corresponding inter-LL transition energy from –(N-1) to N is:

$$E(\pm k_w)^{-(N-1)\to N} = \sqrt{\frac{4a^2}{l_B^4}N^2 + \frac{2\hbar^2 {v_F}^2}{l_B^2}N} + \sqrt{\frac{4a^2}{l_B^4}(N-1)^2 + \frac{2\hbar^2 {v_F}^2}{l_B^2}(N-1)}\,. \tag{6}$$

Since the Hamiltonian is still particle-hole symmetric, the inter-LL transition from -N to (N-1) will be the same energy. This analytical solution only exists when magnetic field is applied along the Weyl node separation direction. For a magnetic field point to an arbitrary direction in ab plane, one can utilize an effective Hamiltonian reduced from the two-band model near each Weyl points. The Hamiltonian describing a single Weyl cone near $k_z=\pm k_w$ is:

$$H(k) = -ak^2\sigma_z + \hbar v_F k \cdot \sigma\,, \tag{7}$$

As a result, the LL energy shares the same form as the two-band model at $k_z=k_w$:

$$E^0 = -\frac{a}{l_B^2}\,,\ \text{N=0} \tag{8}$$

$$E^N = -\frac{a}{l_B^2} \pm \sqrt{(\frac{2a}{l_B^2}N)^2 + \frac{2\hbar^2 {v_F}^2}{l_B^2}N}\,,\ \text{N}\geq 1 \tag{9}$$

as well as the inter-LL transition energy:

$$E^{-(N-1)\to N} = \sqrt{\frac{4a^2}{l_B^4}N^2 + \frac{2\hbar^2 {v_F}^2}{l_B^2}N} + \sqrt{\frac{4a^2}{l_B^4}(N-1)^2 + \frac{2\hbar^2 {v_F}^2}{l_B^2}(N-1)}\,. \tag{10}$$

Since this single Weyl point Hamiltonian is symmetric along k_x, k_y, k_z, the actual form of the inter-LL transition does not depend on the orientation of the magnetic field direction. In particular, the inter-band transitions we are interested in are at/near the Weyl points, so eq. S10 will still be valid for fitting the data sets with magnetic field 22 deg and 45 deg to the a-axis. In addition, if we consider the particle-hole asymmetry, the Hamiltonian can be further modified as:

$$H(k) = \frac{\hbar^2 k^2}{2m^*} - ak^2\sigma_z + \hbar v_F k \cdot \sigma\,, \tag{11}$$

which will give a small but finite energy splitting between the –(N-1)→N and -N→(N-1) transitions.

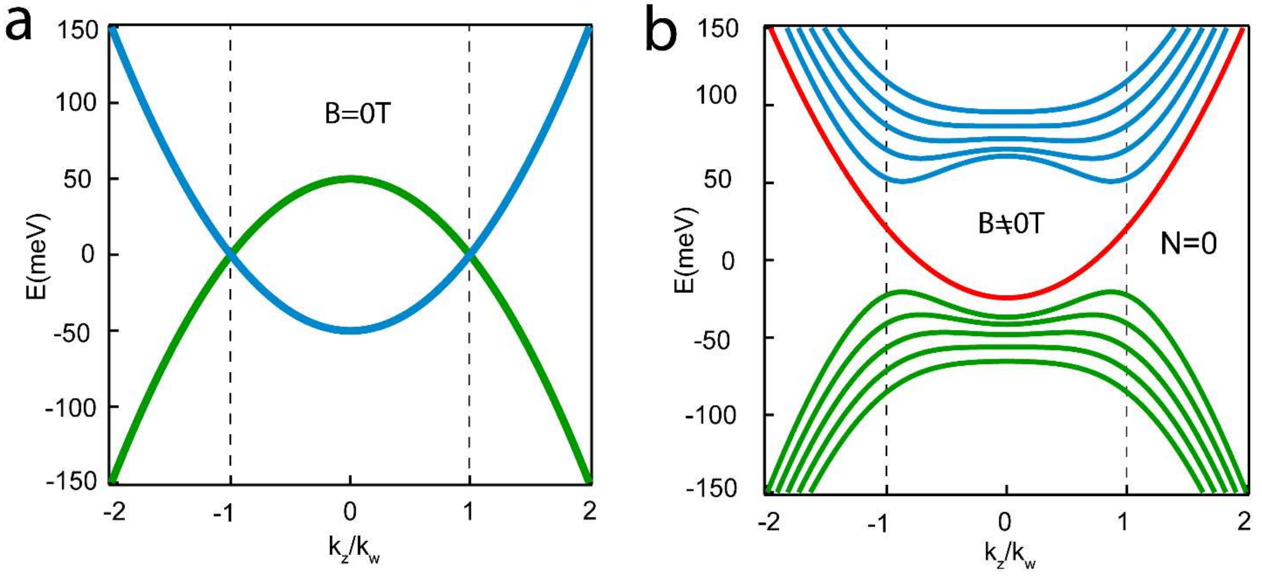

Fig. S1 Low energy dispersion along k_z with the two-band model. a. *Dispersion relation at zero magnetic field, Weyl nodes are sitting at $k_z=\pm k_w$, labeled with dashed lines.* ***b.*** *Landau levels at a finite magnetic field along z direction of both Weyl nodes. The N=0 Landau band is labeled in red.*

2. Anisotropy of the W2 pockets

Assuming that the pockets have two-fold rotation symmetry, like in an ellipse, a magnetic field pointing along either the 45° or 0° directions will result in two different cross-sections for the pockets. If the ellipse is elongated along the a/b axis, at $\theta = 45°$, all W2 pockets will have the same cross-section (c_{45})—resulting in one inter LL transition energy for one branch. At $\theta = 0°$, half of the W2 pockets will have a smaller (bigger) cross-section than that at $\theta = 45°$, resulting in two different inter LL transition energies in the same branch that sandwich the transition energy at theta=45° (Fig. S2b). However, we did not observe such structures in Fig. 4a: instead, we see only a continuous redshift of the lines as we rotate from $\theta = 45°$ to $\theta = 0°$. Note that this is distinct from the small splitting we show in Figure 4c that may arise due to particle-hole asymmetry. Other possible Fermi surface shapes with two-fold rotation symmetry will give similar results and also do not agree with the data: in general, two-fold Fermi surfaces will always produce a feature that red-shifts when rotating away from $\theta = 0°$, which we do not observe.

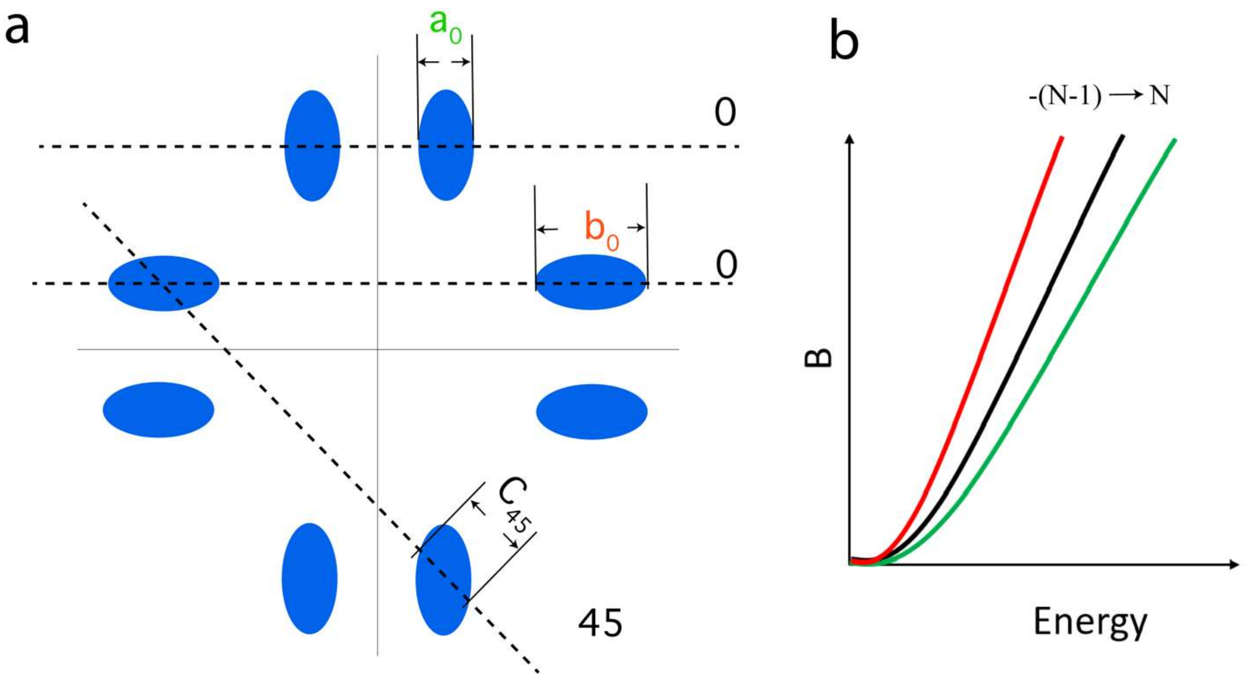

Fig. S2 Schematic of anisotropic W2 pockets. ***a.*** *The W2 Fermi surface, with each pocket having only C2 symmetry.* a_0 *and* b_0 *indicates the cross-section when magnetic field is aligned with a/b axis.* c_{45} *is the cross-section when magnetic field is 45 deg to the a/b axis.* ***b.*** *Schematic of the inter LL transitions for magnetic field aligned with a/b axis (green and red, respectively) and 45 deg to either axis (black). The LL transition for the 45 deg case is sandwiched between the two other transitions, which is not what we observe in Figure 4a.*

3. Fermi energy correction induced by nonlinear dispersion

By considering the contribution from the non-linear dispersion, the Fermi energy can be estimated from: $E = E_F(1 + \frac{\sqrt{N-1+0.036(N-1)^2}}{\sqrt{N+0.036N^2}})$. Taking the -1→2 transition as an example gives a 0.05 meV correction for the Fermi energy which is smaller than the measurement uncertainty.

4. Estimation of plasmon-induced energy shift

In Voigt geometry, the transition frequencies will be shifted due to coupling with the plasmon and the actual resonance will occur at $\omega = \sqrt{\omega_0^2 + \omega_p^2}$ instead of ω_0, where $\hbar\omega_0$ is the transition energy without coupling

to the plasmon and ω_p is the plasmon frequency *(34)*. In a 3-dimenonal Weyl system, where the energy dispersion is linear, the plasmon frequency at zero temperature and finite density is proportional to the Fermi energy and $n^{1/3}$, where n is the carrier density *(35)*. The form of the plasmon frequency can be written as $\omega_p = \sqrt{\frac{2g\alpha}{3\pi\kappa_0}}E_F$,where $\alpha = \frac{k_e e^2}{\hbar v_F k}$ is a dimensionless constant (the ratio between coulomb and kinetic energy), g is the degeneracy of the Weyl nodes, and E_F is Fermi energy. The parameter κ_0=1 + $\frac{g\alpha}{3\pi} ln\frac{\Lambda_L}{2k_F}$, where $\Lambda_L = k_c e^{\frac{g\alpha}{3\pi}}$, is known as the Landau pole *(36)*, and k_c is the cut-off corresponding to the momentum scale above which the dispersion deviates from linear. For Weyl semimetals like TaAs, with Weyl pairs in close proximity in the Brillouin zone, the Landau pole could be very small *(37)*. Therefore, the estimated energy of the plasmon is $\hbar\omega_p \approx$ 1.5meV, which is much smaller than the transition energies we observed (>10 meV). Since the plasmon induced resonance frequency shift is less than few percent *(38)*, which is within the error bar of the data fitting in Figure 2.b, the effects induced by plasmon can be neglected.

5. Inter-LL transition assignment and fine features

Following the derivation of the optical selection rules *(18,39)*, the inter-LL transitions with Δ|N| = ±1 or Δ|N| = 0 are associated to light polarization perpendicular or parallel to the *B* field. In the experimental data, the main branches (Fig. 1d and Fig. 2a) are attributed to the transitions between Δ|N| = ±1 LLs at the W2 Weyl points. Using the Hamiltonian described above, we can reproduce the dispersions with Fermi velocity 2.2×10^5 m/s for all branches. The four major transitions traced by dashed lines in Fig. 1d and 2a agreed well with the calculated *Δ*|*N*|= ±1 transitions. Other than these strong features, a set of branches with weaker intensity are likely to be the Δ|N| = 0 transitions, which are excited by light polarization parallel to the B field. By using the same fitting parameters, we can calculate the transition energies for the Δ|N| = 0 transitions, which agree with the experimental data very well (Fig. S3a). If we compare the energies of the strong features with the calculated Δ|N| = 0 transitions, they will not agree well even after optimizing the fitting parameters (Fig. S3b). Similarly, calculated Δ|N| = ±1 transition energies do not agree with the weaker branches (Fig. S3c). As a result, we assign the stronger branches and weaker branches to the to Δ|N| = ±1 and Δ|N| = 0 inter-LL transitions, respectively.

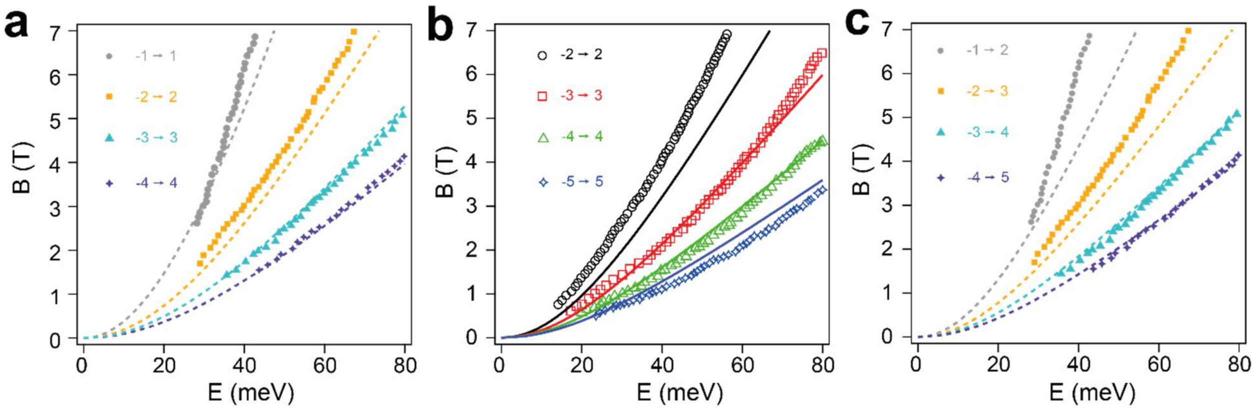

Fig. S3 Fitting of the stronger and weaker features with different assignment of the inter-LL transitions. ***a.*** *Comparison between the energies of weaker features extracted from Fig. 1d and calculated inter-LL transition energies of* $\Delta|N| = 0$ *with the same fitting parameters as used in the main text. The four branches of the weaker features agree well with the calculated energies plotted in grey, orange, cyan and purple dashed lines*. ***b.*** *Assigning the main features shown in Fig.1d to* $\Delta|N| = 0$ *transitions and comparing with the calculated inter-LL transition energies. The solid lines in black, red, green and blue are the best fit to the data with a Fermi velocity of* 1.9×10^5 m/s. ***c.*** *Fitting the weaker features to* $\Delta|N| = \pm1$ *transitions with the same parameter as used in Fig. S3b. From these fits it is clear that the assignments of the strong features to* $\Delta|N| = 0$ *transitions and the weak features to* $\Delta|N| = \pm1$, *does not produce as good of fits as the opposite assignment (which is the assignment used in the main text)*.

Ideally, with perfectly unpolarized incidence light, the intensity of both $\Delta|N| = \pm1$ and $\Delta|N| = 0$ transitions should be comparable following the derivation of the matrix elements *(18,39)*. However, in the experimental data, the features correspond to $\Delta|N| = 0$ transitions are weaker than those from $\Delta|N| = \pm1$. A possible reason could be that although the incident light is largely unpolarized, the experimental apparatus could prefer one polarization more than the other due to the geometry of components and the sample. In our case, we suspect that light with polarization perpendicular to B is stronger than the light with polarization parallel to B, which results in the intensity difference of the two set of features.

6. Normalized reflection spectra

Fig. S4 shows part of normalized reflection spectra that were used to generate Fig. 1d in the main text.

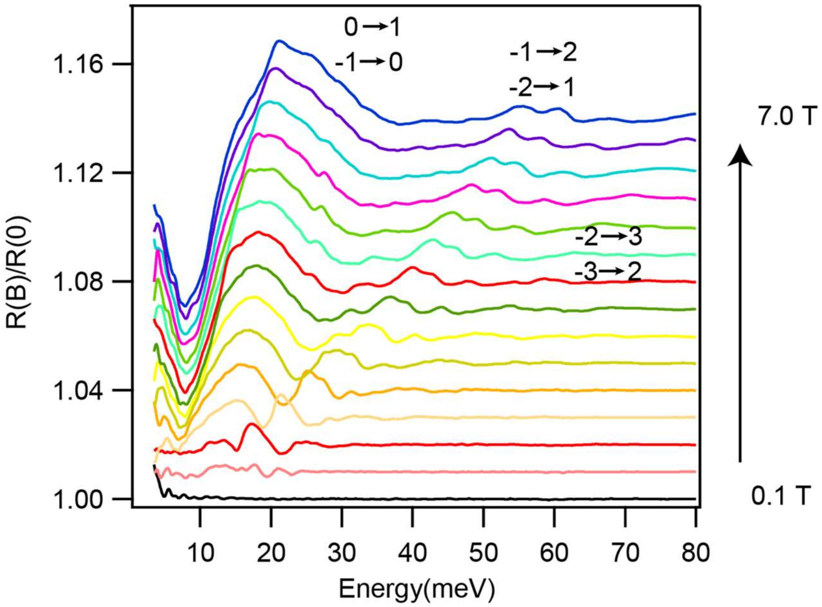

Fig. S4 Reflection spectra normalized to zero field, R(B)/R(0), from 0.1 T to 7 T. *The labels indicate the corresponding inter-LL transitions. The spectra are vertically shifted for clarity.*

7. Estimation of the Energy scale of W2 Weyl cone

To estimate the energy scale of the W2 Weyl cone, we compared the fitting results based on the phenomenological model described in SI section 1, which includes deviations from the linear dispersion and incorporates the saddle points and the $B^{0.62}$ scaling. The data, including transitions at a higher energy at up to 17.5T, is shown in Figure. S5. The solid lines are fits that result from the phenomenological model with the same set of parameters mentioned in the main text. The dashed lines are calculated energy dispersion based on $B^{0.62}$ scaling. As we can see, below 80-90 meV, the data follows very well with both $B^{0.62}$ scaling and the phenomenological model. However, above 100meV, the magnetic field dependence of the transition energy deviates from $B^{0.62}$, while the full model continues to capture the dispersion relatively well. Such deviation from the simple scaling could be an indication that the energy scale is approaching the saddle point. In addition, we can calculate the energy scale of the saddle point from the phenomenological model with the extracted fitting parameters (a=0.5 eV nm^2). If we take k_w = 0.5 nm^{-1} from Ref. *(4)*, the

energy of the saddle point is roughly 120 meV. Consequently, the energy scale of about 120 meV agrees with the theoretical prediction of W2 in Ref. *(8)*.

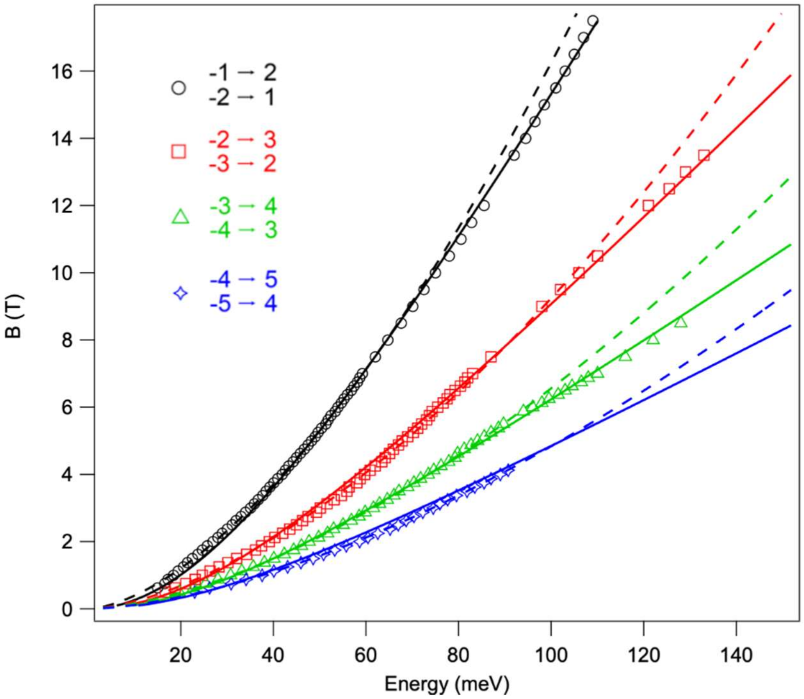

Fig. S5 The Δ|N| = ±1 transitions up to 17.5 T. *The markers are LL-transitions extracted from the normalized reflection spectra. The dashed lines represent the dispersion based on the* $B^{0.62}$ *scaling. The solid lines are fits to the model described in section 1. Above 100meV, the magnetic field dependence of the transition energy deviates from* $B^{0.62}$ *but continues to fit the phenomenological model.*

8. Pulse echo ultrasound

A longitudinal thin-film ZnO transducer was reactively sputtered onto the flat a-b plane surface shown in Figure 1b of the main text. The relative change in the c-axis sound velocity was measured as a function of magnetic field with a digital pulse-echo technique described in Ref. *(20)*.

Fig. S6a shows quantum oscillations in the c-axis sound velocity for magnetic field applied along the [100] direction, corresponding to $\theta = 0°$. The oscillations are highly asymmetric around the background – either there is a very low frequency oscillation that is hard to resolve because of the dominant F = 1.4 T oscillation, or there is a non-trivial background due to movement of the chemical potential with field. Fig. S6b shows the Fourier transform of these data. The dominant peak at F = 1.4 T corresponds to the oscillations most visible by eye in panel a. The peak near F = 0.8 T is hard to resolve separately from the background at lower frequency, but is likely the source of asymmetry seen in panel a. This peak corresponds well with the quantum limit measured at the W2 Weyl points using magneto-infrared spectroscopy, but it is impossible

to say anything conclusive about the Fermi surface using the quantum oscillation data alone because the very low frequency is difficult to distinguish from the background.

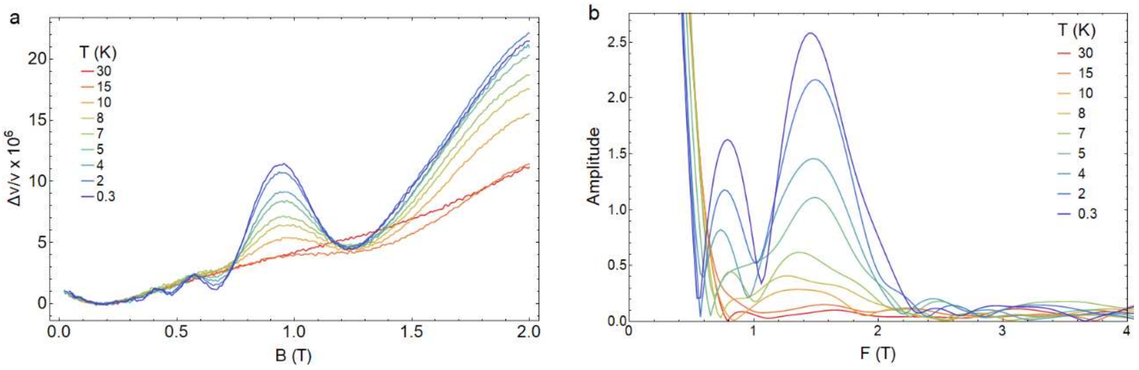

Fig. S6 Change in the c-axis sound velocity with magnetic field applied along the [100] direction. a. *The raw data measured at 802 MHz. Oscillations can be seen as low as 0.2 T, indicating high sample quality.* ***b.*** *Fourier transform of the data from the left panel between 0.24 and 2 Tesla. The large upturn at low frequency is due to the background, which has not been removed.*

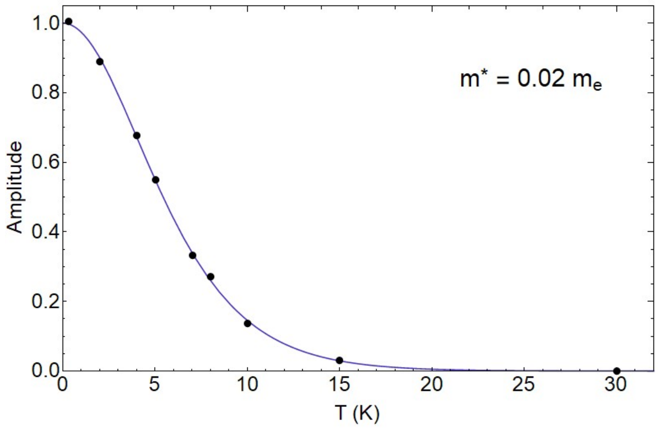

Fig. S7 Lifshitz-Kosevich mass plot. *The amplitude is defined as the maximum of Δv/v at approximately 0.9 Tesla minus the minimum at approximately 0.7 Tesla. The fit is to the standard LK expression,* $A(T) = X/\sinh X$, *where* $X = \frac{2\pi^2 k_B T}{\hbar\omega_c}$, *and* $\omega_c = \frac{eB}{m^*}$ *is the cyclotron frequency.*

Fig. S7 shows the quantum oscillation amplitude of the 1.4 T frequency as a function of temperature, yielding a cyclotron effective mass of $m^* = 0.02\ m_e$. The frequency and mass combine to give a Fermi velocity of 3.8x10^5 m/s – nearly a factor of 2 higher than what we measure at the W2 Weyl point in the infrared spectroscopy.

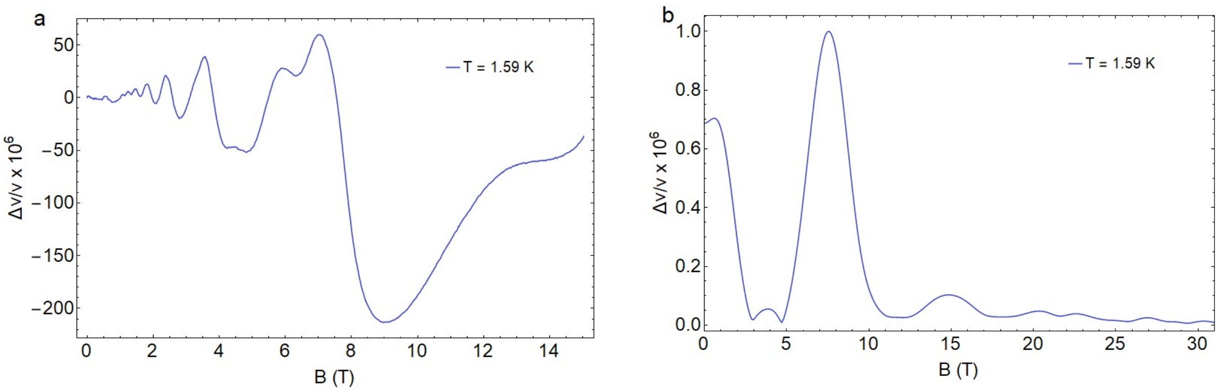

Fig. S8 Change in the c-axis sound velocity with magnetic field applied along the [001] direction. a. *Raw oscillatory data taken at the same ultrasonic frequency and with the same method as in Fig. S6 but for field along the c axis.* ***b.*** *Fourier transform of the data in a), showing a clear peak at F = 7.5 T, in agreement with the Fermi surface area of the W1 pocket measured previously (19).*

Figure S8 shows quantum oscillations in the sound velocity measured for field along the c axis. The main frequency of approximately 7.5 Tesla is consistent with what has been identified in other studies as belonging to the Fermi surface around the W1 Weyl node *(19,22)*.

REFERENCES AND NOTES

1. B. Yan, C. Felser, Topological Materials: Weyl Semimetals. *Annu. Rev. Condens. Matter Phys.* **8**, 337–354 (2017).

2. A. Bansil, H. Lin, T. Das, Colloquium: Topological band theory. *Rev. Mod. Phys.* **88**, 21004 (2016).

3. N. P. Armitage, E. J. Mele, A. Vishwanath, Weyl and Dirac semimetals in three-dimensional solids. *Rev. Mod. Phys.* **90**, 015001 (2018).

4. S.-Y. Xu, I. Belopolski, N. Alidoust, M. Neupane, G. Bian, C. Zhang, R. Sankar, G. Chang, Z. Yuan, C.-C. Lee, S.-M. Huang, H. Zheng, J. Ma, D. S. Sanchez, B. Wang, A. Bansil, F. Chou, P. P. Shibayev, H. Lin, S. Jia, M. Z. Hasan, Discovery of a Weyl fermion semimetal and topological Fermi arcs. *Science* **349**, 613–617 (2015).

5. B. Q. Lv, H. M. Weng, B. B. Fu, X. P. Wang, H. Miao, J. Ma, P. Richard, X. C. Huang, L. X. Zhao, G. F. Chen, Z. Fang, X. Dai, T. Qian, H. Ding, Experimental discovery of Weyl Semimetal TaAs. *Phys. Rev. X* **5**, 31013 (2015).

6. H. Weyl, Elektron und Gravitation. I. *Zeitschrift für Phys.* **56**, 330–352 (1929).

7. Y. Li, F. D. M. Haldane, Topological nodal cooper pairing in doped Weyl metals. *Phys. Rev. Lett.* **120**, 067003 (2018).

8. D. Grassano, O. Pulci, A. Mosca Conte, F. Bechstedt, Validity of Weyl fermion picture for transition metals monopnictides TaAs, TaP, NbAs, and NbP from ab initio studies. *Sci. Rep.* **8**, 3534 (2018).

9. C.-C. Lee, S.-Y. Xu, S.-M. Huang, D. S. Sanchez, I. Belopolski, G. Chang, G. Bian, N. Alidoust, H. Zheng, M. Neupane, B. Wang, A. Bansil, M. Z. Hasan, H. Lin, Fermi surface interconnectivity and topology in Weyl fermion semimetals TaAs, TaP, NbAs, and NbP. *Phys. Rev. B* **92**, 235104 (2015).

10. S. Kimura, H. Yokoyama, H. Watanabe, J. Sichelschmidt, V. Süß, M. Schmidt, C. Felser, Optical signature of Weyl electronic structures in tantalum pnictides Ta *Pn* (*Pn* = P, As). *Phys. Rev. B* **96**,

075119 (2017).

11. S.-B. Zhang, H.-Z. Lu, S.-Q. Shen, Linear magnetoconductivity in an intrinsic topological Weyl semimetal. *New J. Phys.* **18**, 53039 (2016).

12. H.-Z. Lu, S.-B. Zhang, S.-Q. Shen, High-field magnetoconductivity of topological semimetals with short-range potential. *Phys. Rev. B* **92**, 45203 (2015).

13. M. Orlita, D. M. Basko, M. S. Zholudev, F. Teppe, W. Knap, V. I. Gavrilenko, N. N. Mikhailov, S. A. Dvoretskii, P. Neugebauer, C. Faugeras, A.-L. Barra, G. Martinez, M. Potemski, Observation of three-dimensional massless Kane fermions in a zinc-blende crystal. *Nat. Phys.* **10**, 233–238 (2014).

14. Y. Jiang, Z. Dun, S. Moon, H. Zhou, M. Koshino, D. Smirnov, Z. Jiang, Landau quantization in coupled Weyl points: A case study of Semimetal NbP. *Nano Lett.* **18**, 7726–7731 (2018).

15. X. Yuan, Z. Yan, C. Song, M. Zhang, Z. Li, C. Zhang, Y. Liu, W. Wang, M. Zhao, Z. Lin, T. Xie, J. Ludwig, Y. Jiang, X. Zhang, C. Shang, Z. Ye, J. Wang, F. Chen, Z. Xia, D. Smirnov, X. Chen, Z. Wang, H. Yan, F. Xiu, Chiral Landau levels in Weyl semimetal NbAs with multiple topological carriers. *Nat. Commun.* **9**, 1854 (2018).

16. S. Kimura, Y. Yokoyama, Y. Nakajima, H. Watanabe, J. Sichelschmidt, V. Süß, M. Schmidt, C. Felser, in *Proceedings of the International Conference on Strongly Correlated Electron Systems* (*SCES2019*) (Journal of the Physical Society of Japan, 2020), vol. 30 of *JPS Conference Proceedings*.

17. S. Polatkan, M. O. Goerbig, J. Wyzula, R. Kemmler, L. Z. Maulana, B. A. Piot, I. Crassee, A. Akrap, C. Shekhar, C. Felser, M. Dressel, A. V. Pronin, M. Orlita, Magneto-optics of a Weyl semimetal beyond the conical band approximation: Case study of TaP. *Phys. Rev. Lett.* **124**, 176402 (2020).

18. A. L. Levy, A. B. Sushkov, F. Liu, B. Shen, N. Ni, H. D. Drew, G. S. Jenkins, Optical evidence of the chiral magnetic anomaly in the Weyl semimetal TaAs. *Phys. Rev. B.* **101**, 125102 (2020).

19. B. J. Ramshaw, K. A. Modic, A. Shekhter, Y. Zhang, E.-A. Kim, P. J. W. Moll, M. D. Bachmann,

M. K. Chan, J. B. Betts, F. Balakirev, A. Migliori, N. J. Ghimire, E. D. Bauer, F. Ronning, R. D. McDonald, Quantum limit transport and destruction of the Weyl nodes in TaAs. *Nat. Commun.* **9**, 2217 (2018).

20. X. Huang, L. Zhao, Y. Long, P. Wang, D. Chen, Z. Yang, H. Liang, M. Xue, H. Weng, Z. Fang, X. Dai, G. Chen, Observation of the Chiral-Anomaly-Induced Negative Magnetoresistance in 3D Weyl Semimetal TaAs. *Phys. Rev. X* **5**, 31023 (2015).

21. F. Arnold, M. Naumann, S.-C. Wu, Y. Sun, M. Schmidt, H. Borrmann, C. Felser, B. Yan, E. Hassinger, Chiral Weyl pockets and fermi surface topology of the Weyl Semimetal TaAs. *Phys. Rev. Lett.* **117**, 146401 (2016).

22. C.-L. Zhang, B. Tong, Z. Yuan, Z. Lin, J. Wang, J. Zhang, C.-Y. Xi, Z. Wang, S. Jia, C. Zhang, Signature of chiral fermion instability in the Weyl semimetal TaAs above the quantum limit. *Phys. Rev. B* **94**, 205120 (2016).

23. P. J. W. Moll, A. C. Potter, N. L. Nair, B. J. Ramshaw, K. A. Modic, S. Riggs, B. Zeng, N. J. Ghimire, E. D. Bauer, R. Kealhofer, F. Ronning, J. G. Analytis, Magnetic torque anomaly in the quantum limit of Weyl semimetals. *Nat. Commun.* **7**, 12492 (2016).

24. K. A. Modic, T. Meng, F. Ronning, E. D. Bauer, P. J. W. Moll, B. J. Ramshaw, Thermodynamic signatures of Weyl Fermions in NbP. *Sci. Rep.* **9**, 2095 (2019).

25. M. M. Jadidi, M. Kargarian, M. Mittendorff, Y. Aytac, B. Shen, J. C. König-Otto, S. Winnerl, N. Ni, A. L. Gaeta, T. E. Murphy, H. D. Drew, Nonlinear optical control of chiral charge pumping in a topological Weyl semimetal. *Phys. Rev. B.* **102**, 245123 (2020).

26. H. B. Nielsen, M. Ninomiya, The Adler-Bell-Jackiw anomaly and Weyl fermions in a crystal. *Phys. Lett. B* **130**, 389–396 (1983).

27. V. Aji, Adler-Bell-Jackiw anomaly in Weyl semimetals: Application to pyrochlore iridates. *Phys. Rev. B* **85**, 241101 (2012).

28. A. A. Burkov, Chiral anomaly and transport in Weyl metals. *J. Phys. Condens. Matter* **27**, 113201

(2015).

29. D. T. Son, B. Z. Spivak, Chiral anomaly and classical negative magnetoresistance of Weyl metals. *Phys. Rev. B* **88**, 104412 (2013).

30. F. Arnold, C. Shekhar, S.-C. Wu, Y. Sun, R. D. dos Reis, N. Kumar, M. Naumann, M. O. Ajeesh, M. Schmidt, A. G. Grushin, J. H. Bardarson, M. Baenitz, D. Sokolov, H. Borrmann, M. Nicklas, C. Felser, E. Hassinger, B. Yan, Negative magnetoresistance without well-defined chirality in the Weyl semimetal TaP. *Nat. Commun.* **7**, 11615 (2016).

31. T. Schumann, M. Goyal, D. A. Kealhofer, S. Stemmer, Negative magnetoresistance due to conductivity fluctuations in films of the topological semimetal Cd_3As_2. *Phys. Rev. B* **95**, 241113 (2017).

32. Q. Ma, S.-Y. Xu, C.-K. Chan, C.-L. Zhang, G. Chang, Y. Lin, W. Xie, T. Palacios, H. Lin, S. Jia, P. A. Lee, P. Jarillo-Herrero, N. Gedik, Direct optical detection of Weyl fermion chirality in a topological semimetal. *Nat. Phys.* **13**, 842–847 (2017).

33. S.-Y. Xu, I. Belopolski, D. S. Sanchez, M. Neupane, G. Chang, K. Yaji, Z. Yuan, C. Zhang, K. Kuroda, G. Bian, C. Guo, H. Lu, T.-R. Chang, N. Alidoust, H. Zheng, C.-C. Lee, S.-M. Huang, C.-H. Hsu, H.-T. Jeng, A. Bansil, T. Neupert, F. Komori, T. Kondo, S. Shin, H. Lin, S. Jia, M. Z. Hasan, Spin Polarization and texture of the fermi arcs in the Weyl Fermion Semimetal TaAs. *Phys. Rev. Lett.* **116**, 96801 (2016).

34. Palik & Furdyna, J. K. Infrared and microwave magnetoplasma effects in semiconductors. *Reports Prog. Phys.* **33**, 1193–1322 (1970).

35. S. Das Sarma, E. H. Hwang, Collective modes of the massless dirac plasma. *Phys. Rev. Lett.* **102**, 206412 (2009).

36. J. Hofmann, E. Barnes, S. Das Sarma, Interacting Dirac liquid in three-dimensional semimetals. *Phys. Rev. B* **92**, 45104 (2015).

37. J. Hofmann, S. Das Sarma, Plasmon signature in Dirac-Weyl liquids. *Phys. Rev. B* **91**, 241108

(2015).

38. J. Hofmann, Quantum oscillations in Dirac magnetoplasmons. *Phys. Rev. B* **10**, 245140 (2019).

39. J. M. Shao, G. W. Yang Magneto-optical conductivity of Weyl semimetals with quadratic term in momentum. *AIP Adv.* **6**, (2016).
